# Systematic reviews of prognosis studies: a critical appraisal of five core clinical journals

**DOI:** 10.1186/s41512-017-0008-z

**Published:** 2017-03-16

**Authors:** Davide Matino, Chatree Chai-Adisaksopha, Alfonso Iorio

**Affiliations:** 1 Department of Health Research Methods, Evidence, and Impact, McMaster University, Hamilton, Canada; 2 Department of Experimental Medicine, University of Perugia, Perugia, Italy; 3 Department of Medicine, McMaster University, Hamilton, Canada

**Keywords:** Systematic review, Prognosis, MOOSE, PRISMA

## Abstract

**Background:**

Prognosis research refers to the investigation of association between a baseline health state, patient characteristic and future outcomes. The findings of several prognostic studies can be summarized in systematic reviews (SRs), but some characteristics of prognostic studies may result in difficulties when performing the analyses. This study aimed to investigate trends in the volume and quality of SRs of prognostic studies in the literature.

**Methods:**

We conducted a systematic review in five high-impact clinical journals (Annals of Internal Medicine, BMJ, Circulation, JAMA, and Stroke) to identify SRs of prognosis studies focused on fundamental prognosis research and prognostic factor research published between 2000 and 2012. We excluded studies of clinical prediction guides or implementation studies. The quality of the SRs was rated based on the Meta-analysis of Observational Studies in Epidemiology (MOOSE) and the PRISMA checklists.

**Results:**

Over the 13-year period, 1065 SRs were published. Of these, 198 were SRs of prognosis studies. The proportion of all SRs to published articles increased from 0.86% in 2000 to 4.2% in 2012. Likewise, the proportion of prognosis SRs to all SRs increased from 10.3% in 2000 to 17.7% in 2012. MOOSE and PRISMA mean summary scores consistently increased over time for all journals, indicating that the quality of reporting in these SRs has steadily improved. However, several items were not consistently well reported by investigators.

**Conclusions:**

This study shows that there is a growing number of SRs of prognosis studies. However, the quality is suboptimal when assessed with the generic reporting guidelines for observational studies. New reporting guidelines and risk of bias tools for prognosis studies are needed to improve the quality of future research in this field.

**Electronic supplementary material:**

The online version of this article (doi:10.1186/s41512-017-0008-z) contains supplementary material, which is available to authorized users.

## Background

Broadly speaking, prognosis research focuses on the description and prediction of future outcomes in people with a given baseline health state [1]. Its aim is to understand and improve outcomes in people with a specific disease or health condition and to provide evidence for improving healthcare and public health policy. As such, prognosis research can provide important information to support clinical decision-making, the definition of risk groups, and more accurate prediction of disease outcomes [2]. It can help to extend or revise definitions of disease, while identifying unanticipated benefits or harms of healthcare interventions and the need for new interventions to improve patient outcomes [3]. It also has an important role in helping patients to make healthcare-related decisions and planning their lives based on their preferences and reliable evidence [4].

The purpose of this study was to assess the quality of SRs of prognostic studies published over the last decade in five high-impact journals. The prognosis research strategy (PROGRESS) group suggests that prognosis research can be generally classified into four categories:

1. Fundamental prognosis research (to describe and explain future outcomes in relation to current diagnostic and treatment practices) [1].
2. Prognostic factor research (to identify factors associated with subsequent clinical outcomes in patients with a particular disease or health condition) [5].
3. Prognostic model research (to explore the use of combinations of prognostic factors to predict the risk of future clinical outcomes in individual patients) [6].
4. Stratified medicine research (to identify factors that predict patient treatment response, commonly referred to as predictive factors) [7].

This study will focus on the first two categories: fundamental prognosis research to describe and explain outcomes in patients with a given disease or condition and research on prognostic factors associated with outcomes. We chose to focus on these more traditional studies of prognosis because the latter two types (prediction models and stratified medicine research) comprise rapidly expanding and evolving fields and as such are beyond the scope of this study.

SRs aim to summarize the collection of primary studies on a given topic. A common issue when conducting SRs is the limitations of the primary studies included in the review and such is the case in SRs of prognosis studies [8]. Prognosis studies are often too small and too poorly designed and/or analyzed to provide reliable evidence. An increasing body of evidence highlights the limitations of primary studies of prognosis, including those inherent to the retrospective design of many studies, variations in inclusion criteria, and variables included in adjusted analyses, inadequate reporting methods, and differences in how results are reported [9, 10].

SRs can help to identify the strengths and limitations of research in a specific field. The purpose of this study was to identify the number of SRs of prognosis studies published over a 13-year period in five high-impact journals and to assess the quality of these reviews.

## Methods

### Inclusion criteria

SRs of prognosis studies were included if they were summarized primary prognosis study types 1 or 2, as defined by PROGRESS—that is, studies reporting on the overall prognosis of a broad population of patients with a specific health condition, or assessment of one or more prognostic variables. SRs of diagnosis, clinical prediction models, or implementation studies were excluded. We also excluded narrative reviews, and non-systematic reviews published as editorials or letters.

### Literature search

We conducted a Medline search to identify SRs published from 2000 to 2012 in five high-impact journals in internal medicine and cardiovascular disease (Annals of Internal Medicine, BMJ, Circulation, JAMA, and Stroke). Search details are provided in the Appendix.

### Study selection

Pairs of authors independently examined in duplicate the titles and abstracts of retrieved references to identify SRs of prognosis studies fulfilling our inclusion criteria. Disagreements among the authors were resolved by consensus. The full texts of all selected articles were obtained, and pairs of authors examined, in duplicate, the eligibility of each article for inclusion; in cases of disagreement, a third independent reader was involved.

### Quality assessment

The methodologies to conduct SRs of prognosis are not yet fully developed, and no agreement exists on the key features defining good quality research in this field [9, 10]. Therefore, we used the PRISMA (Preferred Reporting Items for Systematic reviews and Meta-Analyses) [11] and the MOOSE (Meta-analysis of Observational Studies in Epidemiology) checklists [12] as a proxy for the quality of the included SRs of prognosis studies. Pairs of researchers assessed each of the included SRs in duplicate using the two checklists. Each item in the checklist was rated defining it as present/absent (or completed/incomplete) or not applicable (NA). In cases of disagreement, a third independent reader was involved.

### Statistical analysis

Summary descriptive statistics were used to report the number of SRs as proportion of all research articles as well as the number of SRs of prognosis as a proportion of all SRs.

The proportion of systematic reviews that met individual items from the MOOSE and PRISMA checklists by year was reported with 95% confidence interval (CIs). Summary scores for each review were calculated as the sum of the scores for each item divided by total number of the items in the checklists. Mean summary scores with 95% CIs were reported separately for MOOSE and PRISMA checklists by journal and year of publication. Higher scores indicated more completed items. The change in mean MOOSE and PRISMA scores by year was calculated using ANOVA tests and linear regression.

## Results

### Bibliometric indexes

**Fig. 1:**
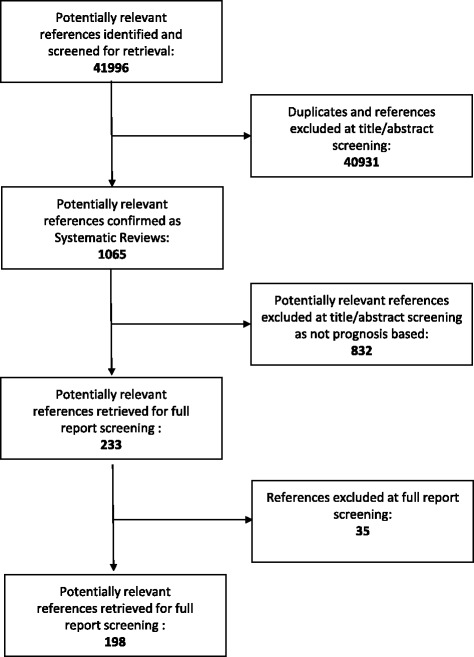
Flow diagram of study selection process

**Fig. 2:**
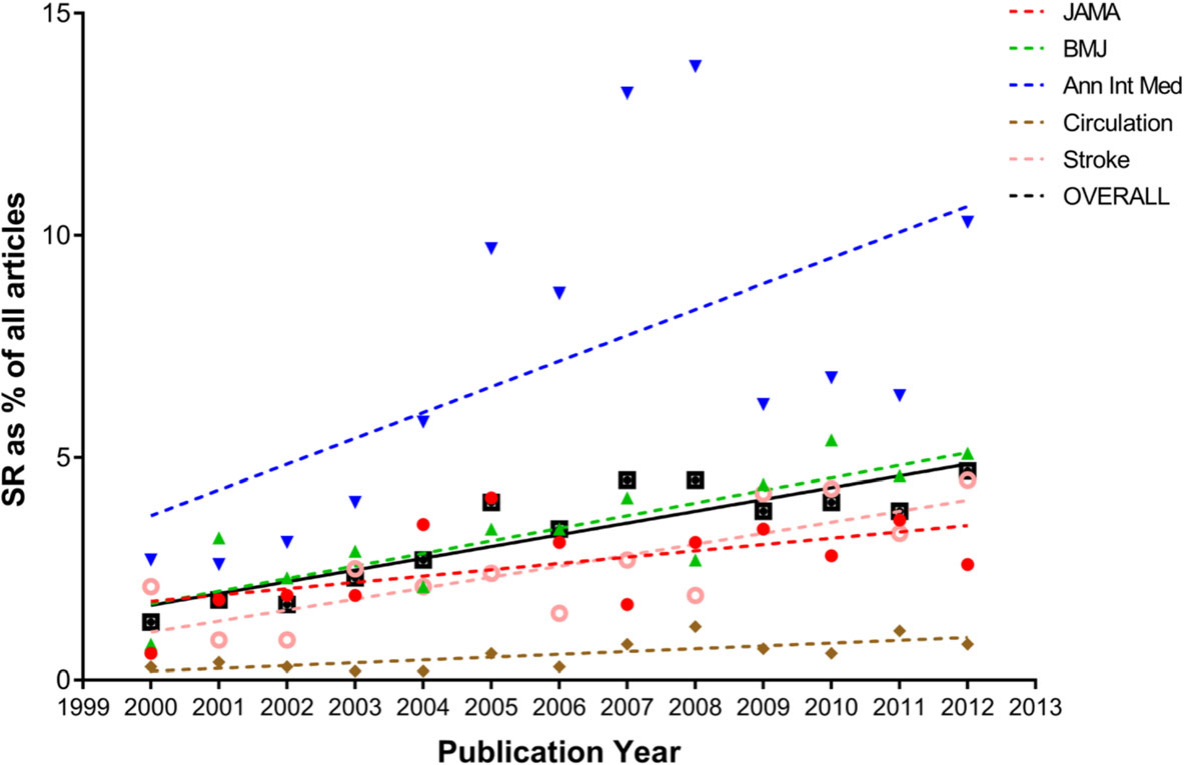
Temporal trend of systematic reviews as a percentage of total published research articles. The figure shows the temporal trend of systematic reviews expressed as percentage of all research articles published in Annals of Internal Medicine, BMJ Circulation, JAMA, and Stroke from 2000 to 2012

**Fig. 3:**
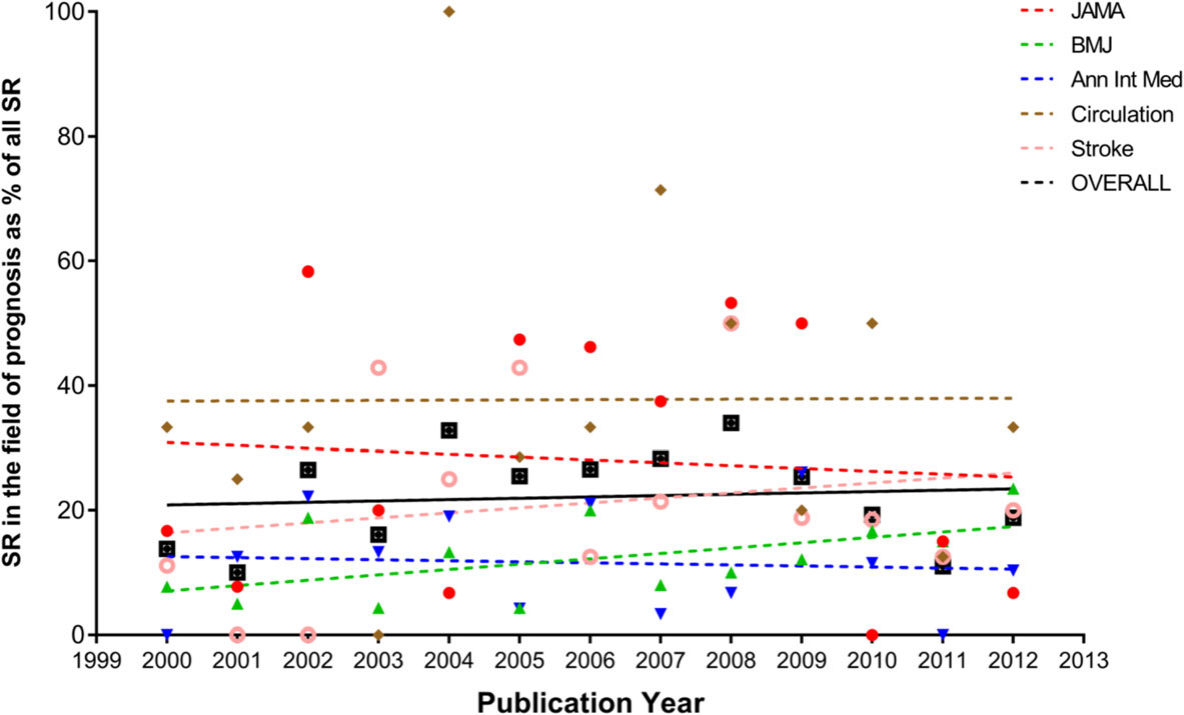
Temporal trend of prognostic systematic reviews as a percentage of total published systematic reviews. The figure shows the temporal trend of prognostic systematic reviews expressed as percentage of all systematic reviews published in Annals of Internal Medicine, BMJ Circulation, JAMA, and Stroke from 2000 to 2012

Over the 13-year search period, 41,996 research articles were published in the five selected journals. One thousand sixty-five SRs were published, of which, 198 met our inclusion criteria (Fig. 1). Figure 2 shows the trend over time for all SRs as a proportion of total research articles, while Fig. 3 shows the trend over time of SRs of prognosis as a proportion of all SRs. Overall, the proportion of all SRs increased from 0.86% in 2000 to 4.2% in 2012. Likewise, the proportion of prognostic SRs to overall SRs slightly increased from 10.3% in 2000 to 17.7% in 2012. Characteristics of the included SRs are reported in Table 1.

**Table 1:** Characteristics of included systematic reviews of prognosis studies

Field	Number of primary studies included in reviews	ANN INT MED	BMJ	CIRCUL	JAMA	STROKE	ALL
Cardiovascular diseases	<10	0	6	3	4	3	16
	11–25	6	4	10	8	2	30
	>25	2	6	7	6	1	22
Neurology^a^ and psychiatry	<10	0	0	0	0	10	10
	11–25	0	5	0	3	18	26
	>25	0	0	1	2	11	14
Oncology	<10	2	0	0	1	0	3
	11–25	1	3	0	1	0	5
	>25	1	1	0	3	0	5
Endocrinology	<10	1	2	2	0	0	5
	11–25	0	1	0	1	0	2
	>25	0	1	1	0	0	2
Other^b^	<10	4	2	1	5	0	12
	11–25	7	6	0	9	0	22
	>25	6	9	0	8	1	24
TOTAL		30	46	25	51	46	198

^a^Includes studies that had non-embolic stroke as a main outcome; embolic stroke has been included under cardiovascular diseases

^b^Includes studies of rheumatology, pediatrics, gastroenterology, geriatrics, nephrology, allergy/immunology, critical care, and infectious diseases

### Quality assessment of SRs of prognosis

Tables 2 and 3 show the proportion of reviews meeting each individual MOOSE and PRISMA item, respectively. The MOOSE and PRISMA items are reported in Additional file 1: Tables S1 and S2. Items from MOOSE that most often were not met were description of study population, qualification of searchers, effort to include all available studies, software used, list of excluded citations with justification, addressing articles published in other languages other than English, methods of handling unpublished studies, assessment of confounding, assessment of study quality and risk of bias, provision of appropriate tables and graphic, and consideration of alternative explanations for the results. Items from PRISMA that most often were not met were description of review protocol, search strategy, method of data extraction, method of assessing risk of bias, method of additional analysis, and presentation of risk of bias.

**Table 2:** Quality assessment of SRs of prognosis based on the MOOSE checklist

Item on MOOSE checklist	Proportion fulfilled	95% C.I.	
		Lower	Upper
Background			
1 - Problem definition	0.99	0.96	1.00
2 - Hypothesis statement	0.97	0.94	0.99
3 - Description of study outcome(s)	0.77	0.71	0.83
4 - Type of exposure or intervention used	0.92	0.87	0.95
5 - Type of study design used	0.88	0.83	0.92
6 - *Study population*	*0.59*	*0.52*	*0.66*
Search strategy			
7 - *Qualification of searchers* (*e.g*., *librarians and investigators*)	*0.31*	*0.25*	*0.38*
8 - Search strategy, including time period included in the synthesis and keywords	0.77	0.70	0.82
9 - *Effort to include all available studies*, *including contact with authors*	*0.53*	*0.46*	*0.59*
10 - Databases and registries searched	0.88	0.83	0.92
11 - *Search software used*, *name and version*, *including special features used*	*0.29*	*0.23*	*0.36*
12 - Use of hand searching (e.g., reference lists of obtained articles)	0.72	0.65	0.78
13 - *List of citations located and those excluded, including justifications*	*0.37*	*0.31*	*0.44*
14 - *Method of addressing articles published in languages other than English*	*0.42*	*0.35*	*0.49*
15 - *Method of handling abstracts and unpublished studies*	*0.28*	*0.22*	*0.34*
16 - *Description of any contact with authors*	*0.42*	*0.36*	*0.49*
Methods			
17 - Description of relevance or appropriateness of studies assembled for assessing the hypothesis to be tested	0.72	0.65	0.78
18 - *Rationale for the selection and coding of data* (*e.g*., *sound clinical principles or convenience*)	*0.69*	*0.63*	*0.75*
19 - *Documentation of how data were classified and coded* (*e.g*., *multiple raters*, *blinding*, *and interrater reliability*)	*0.69*	*0.62*	*0.75*
20 - *Assessment of confounding* (*e.g*., *comparability of cases and controls in studies where appropriate*)	*0.49*	*0.42*	*0.56*
21 - *Assessment of study quality*, *including blinding of quality assessors*; *stratification or regression on possible predictors of study results*	*0.46*	*0.39*	*0.53*
22 - Assessment of heterogeneity	0.84	0.78	0.88
23 - Description of statistical methods (e.g., complete description of fixed or random effects model, justification of whether the chosen models account for predictors of study results, dose-response models, or cumulative meta-analysis) in sufficient detail to be replicated	0.83	0.77	0.88
24 - *Provision of appropriate tables and graphics*	*0.32*	*0.25*	*0.39*
Results			
25 - Graphic summarizing individual study estimates and overall estimates	0.77	0.70	0.82
26 - Table giving descriptive information for each study included	0.84	0.78	0.88
27 - Results of sensitivity testing (e.g., subgroup analysis)	0.74	0.68	0.80
28 - Indication of statistical uncertainty of findings	0.84	0.78	0.88
Discussion			
29 - *Quantitative assessment of bias* (*e.g*., *publication bias*)	*0.54*	*0.47*	*0.60*
30 - *Justification for exclusion* (*e.g*., *exclusion of non-English-language citations*)	*0.41*	*0.34*	*0.48*
31 - *Assessment of quality of included studies*	*0.47*	*0.40*	*0.55*
Conclusions			
32 - *Consideration of alternative explanations for observed results*	*0.64*	*0.57*	*0.71*
33 - Generalization of the conclusions (i.e., appropriate for the data presented and within the domain of the literature review)	0.93	0.88	0.96
34 - *Guidelines for future research*	*0.66*	*0.59*	0.72
35 - Disclosure of funding source	0.80	0.74	0.85

Items in italics are met by <70% of reviews

**Table 3:** Proportion of fulfillment for each individual item of the PRISMA checklist with 95% confidence intervals

Item	Proportion fullfilled	95% C.I.	
		Lower	Upper
Title			
1 - Identify the report as a systematic review, meta-analysis, or both	0.91	0.86	0.95
Abstract			
2 - Provide a structured summary including, as applicable: background; objectives; data sources; study eligibility criteria, participants, and interventions; study eligibility criteria, participants, and interventions; study appraisal and synthesis methods; results; limitations; conclusions and implications of key findings; systematic review registration number	0.91	0.86	0.94
Introduction			
3 - Describe the rationale for the review in the context of what is already known	0.99	0.96	1.00
4 - *Provide an explicit statement of questions being addressed with reference to participants*, *interventions*, *comparisons*, *outcomes*, *and study design* (*PICOS*)	*0.57*	*0.50*	*0.64*
Methods			
5 - *Indicate if a review protocol exists*, *if and where it can be accessed* (*e.g*., *Web address*) *and*, *if available*, *provide registration information including registration number*	*0.11*	*0.08*	*0.17*
6 - Specify study characteristics (e.g., PICOS, length of follow-up) and report characteristics (e.g., years considered, language, publication status) used as criteria for eligibility, giving rationale	0.82	0.76	0.87
7 - Describe all information sources (e.g., databases with dates of coverage, contact with study authors to identify additional studies) in the search and date last searched	0.79	0.72	0.84
8 - *Present full electronic search strategy for at least one database, including any limits used, such that it could be repeated*	*0.58*	*0.51*	*0.65*
9 - State the process for selecting studies (i.e., screening, eligibility, included in systematic review and if applicable included in the meta-analysis)	0.75	0.69	0.81
10 - Describe method of data extraction from reports (e.g., piloted forms, independently, in duplicate) and any processes for obtaining and confirming data from investigators	0.67	0.60	0.74
11 - List and define all variables for which data were sought (e.g., PICOS, funding sources) and any assumptions and simplifications made	0.82	0.76	0.87
12 - *Describe the methods used for assessing risk of bias of individual studies* (*including specification of whether this was done at the study or outcome level*), *and how this information is to be used in any data synthesis*	*0.33*	*0.27*	*0.40*
13 - State the principal summary measures (e.g., risk ration, difference in means)	0.87	0.81	0.91
14 - Describe the methods of handling data and combining results of studies, if done, including measures of for each meta-analysis	0.82	0.76	0.87
15 - *Specify any assessment of risk of bias that may affect the cumulative evidence* (*e.g*., *publication bias*, *selective reporting within studies*)	*0.48*	*0.41*	*0.55*
16 - Describe methods of additional analyses (e.g., sensitivity of analyses, meta-regression) if done, indicating which were pre-specified	0.73	0.66	0.79
Results			
17 - *Give numbers of studies screened*, *assessed for eligibility*, *and included in the review*, *with reasons for exclusions at each stage*, *ideally with a flow diagram*	*0.69*	*0.62*	*0.75*
18 - For each study, present characteristics for which data were extracted (e.g., study size, PICOS, follow-up period) and provide with citations	0.78	0.71	0.83
19 - *Present data on risk of bias for each study and*, *if available*, *any outcome level assessment* (*see item 12*)	*0.28*	*0.23*	*0.35*
20 - For all outcomes considered (benefits or harms) present for each study: (a) simple summary data for each intervention group (b) effect estimates and confidence intervals, ideally with a forest plot	0.77	0.71	0.83
21 - Present results of each meta-analysis done, including confidence intervals and measures of consistency	0.80	0.74	0.85
22 - *Present results of any assessment of risk of bias across studies*	*0.48*	*0.41*	*0.55*
23 - Give results of additional analyses, if done (e.g., sensitivity or subgroup analyses, meta-regression)	0.75	0.68	0.81
Discussion			
24 - Summarize the main findings including the strength of evidence for each main outcome; consider their relevance to key groups (e.g., healthcare providers, users, and policy makers)	0.81	0.75	0.86
25 - Discuss limitations at study and outcome level (e.g., risk of bias) and at review level (e.g., incomplete retrieval of identified research, reporting bias)	0.77	0.70	0.82
26 - Provide a general interpretation of the results in the context of other evidence and implications for future research	0.77	0.71	0.83
Funding			
27 - *Describe the sources of funding for the systematic review and other support* (*e.g*., *supply of data*) *role of founders for the systematic review*	*0.67*	*0.60*	*0.73*

Items in italics are below the proportion score of 0.7

**Fig. 4:**
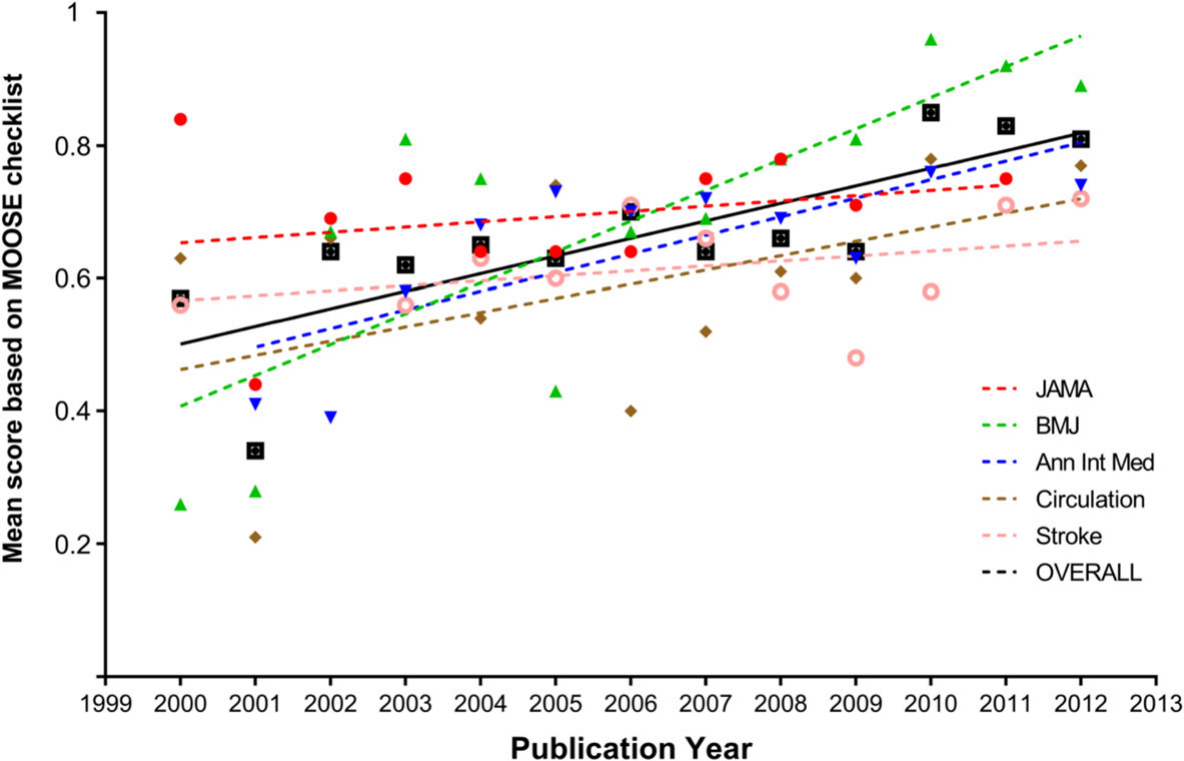
Temporal trend of quality of prognosis systematic reviews based on the MOOSE checklist, expressed as mean summary scores of prognosis systematic reviews published in Annals of Internal Medicine, BMJ Circulation, JAMA, and Stroke from 2000 to 2012

**Fig. 5:**
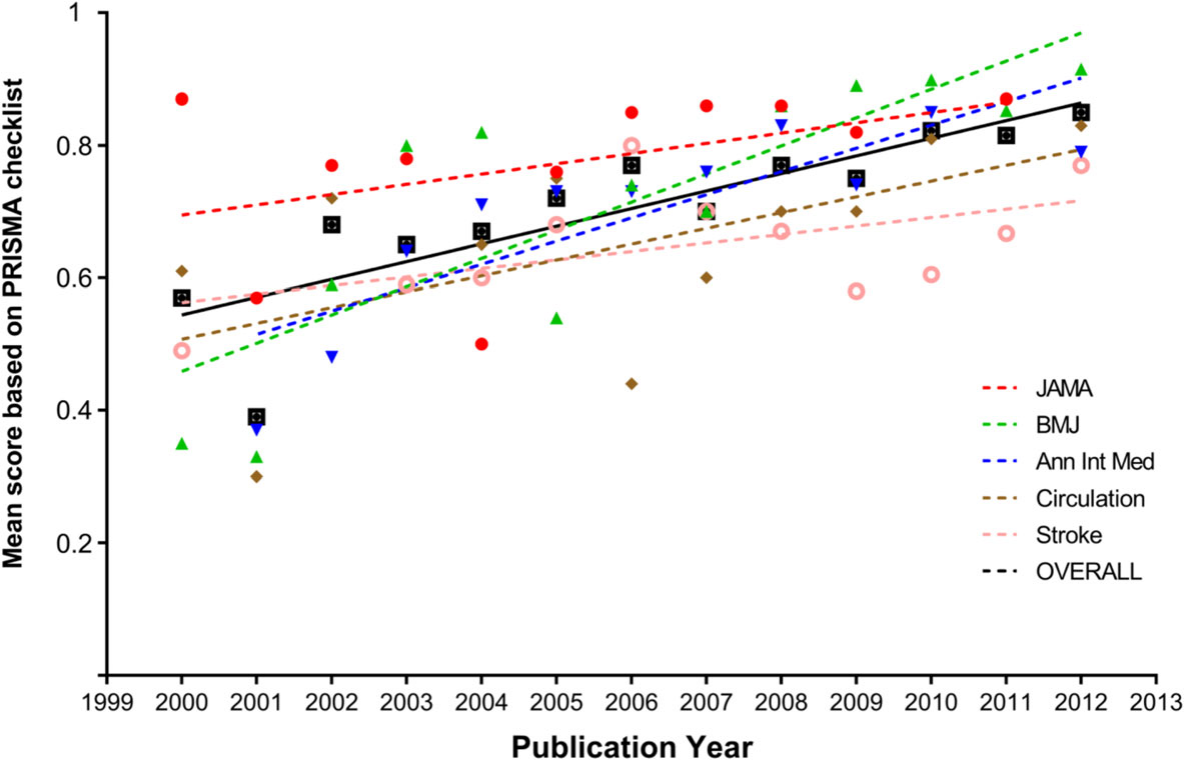
Temporal trend of quality of prognosis systematic reviews based on the PRISMA checklist, expressed as mean summary score in the prognosis systematic reviews published in Annals of Internal Medicine, BMJ Circulation, JAMA, and Stroke in the period 2000–2012

Figures 4 and 5 show by journal the temporal trend of mean summary scores for MOOSE and PRISMA checklists, respectively. Significant differences were found for mean score by year for both MOOSE (*p* = 0.02) and PRISMA (*p* = 0.01). However, a positive correlation was found between year of publication and increase in mean PRISMA score (coefficient = 0.03, 95% CI 0.01 to 0.06, *p* = 0.02) but not for mean MOOSE score (coefficient = 0.02, 95% CI −0.01 to 0.04, *p* = 0.14). The trend over time of mean summary scores was consistently upward for all journals but most evident for BMJ (MOOSE 0.26 [CI 95% 0.12 to 0.39] in 2000 to 0.89 [CI 95% 0.83 to 0.95] in 2012; PRISMA 0.35 [CI 95% 0.19 to 0.51] in 2000 to 0.91 [CI 95% 0.87 to 0.95] in 2012).

## Discussion

In a subset of five high-impact clinical journals, the percentage of published research articles with the Medline publication type (PT) of “Systematic Review” increased five-fold, from 0.86% to 4.2% over 13 years (2000–2012). In the same time frame, the relative percentage of SRs of prognosis remained roughly stable at 20%. As a result, the absolute number of SRs of prognosis has been constantly increasing over time, with a total of 198 published between 2000 and 2012 in JAMA, Annals of Internal Medicine, BMJ, Circulation, and Stroke.

A moderate but progressive improvement in the quality of reporting was observed over time and across the journals considered. However, we found that most of the SRs did not assess the risk for confounding and its possible effect on the direction, strength, and generalizability of the observed association. Confouding may become particularly relevant when prognostic factors are used to tailor treatment decisions to specific populations [13, 14]. We suggest that confounding should be more carefully and systematically evaluated for non-randomized studies assessing prognostic factors, which may require developing dedicated risk of bias assessment tools. Moreover, common mandatory items (e.g., handling of unpublished data, risk of bias assessment, sensitivity analysis.) were poorly reported in SRs of prognostic studies. These limitations should be considered by researchers and reviewers in order to improve the quality of reviews in this field.

We intentionally adopted a non-specific approach to SR appraisal given the various scopes of prognostic research. We focused our study on the first two categories of prognosis research as defined by the PROGRESS group [1] and excluded the latter two categories (clinical prediction models and stratified medicine research). There is increasing information on how and when to perform (or sometimes not perform) a SR of type 1 or 2 studies; summarizing the evidence on clinical prediction models is much more complicated and largely a developing field at this time.

The results presented about assessment and improvement of reporting should be considered in the framework of current research streams in the field. On one side, researchers are focusing on proposing and testing risk of bias assessment tools (QUIPS for studies of risk factors [15] and PROBAST for clinical prediction models, yet unpublished) and specifically on understanding the potential benefits and limitations of SRs and meta-analyses of PROGRESS type 1 (overall prognosis) and PROGRESS type 2 (risk factors) studies [5]. On the other side, GRADE working group members have proposed criteria to assess confidence in SR estimates for type 1 studies, which can improve the use of baseline risk information [2, 4] and of risk factors, which can support planning and execution of subgroup analyses in randomized controlled trials and SRs of randomized controlled trials [15]. In the meantime, it seems appropriate to avoid inundating such a fluidly evolving field [16] with additional ad hoc reporting guidelines, when appropriate use of validated instruments such as MOOSE and PRISMA is far from routine. Eventually, focusing on quality items critical to prognostic research and those that have been difficult to achieve, as shown by our analysis, might be the most efficient way to move forward.

Some limitations of our analysis are worth discussion. First, we limited our sample to five major clinical journals. We cannot extrapolate beyond their content coverage, but we have no reason to expect that important reviews in internal medicine and subspecialties are more likely to be found elsewhere. However, replication of our analyses in different journal sets would be informative. Second, we did not perform a separate analysis of the different types of prognosis studies defined by the PROGRESS group; we cannot draw any conclusions on differences between SR of type 1 and 2 studies. We fully acknowledge the value of the classification in streamlining research in the field, but we consider it premature to look for differences in reporting by study type. In addition, the MOOSE checklist was published in 2000, whereas the PRISMA checklist was not published until 2009. Therefore, it is possible that improvements in the conduct and reporting of SRs might be partially explained by the effects of the publication of PRISMA. Third, we adopted a resource-wise approach and were more interested in appraising the quality of the SRs than the quality and usability of the quality assessment instruments. For this reason, we embedded in our process an initial calibration step to ensure that all the raters were referring to the same scale and were aligned. After that, we rotated all pairs of raters and all raters assessed a similar proportion of articles from each journal. For disagreements, we proceeded immediately to adjudication by a third reviewer. We did not collect information needed to formally calculate a measure of agreement.

## Conclusions

Although many limitations impair the process of evidence synthesis in the field of prognosis, contemporary trends of clinical research toward tailored treatment and patient-centered research make the role of prognosis research even more central. Better evidence relating to disease prognosis will facilitate our understanding of how to move from proof of efficacy to personalized medicine. We found that the quality of SRs of prognosis is suboptimal as assessed using generic reporting guidelines for observational studies. This is in part due to suboptimal reporting, which has been partially reduced over time, and potentially by the imperfect fit of the instruments to assess a very specialized typology of studies. New reporting guidelines and risk of bias tools have been recently (or will soon be) made available for clinical prediction models. Whether these same tools will be suitable to improve the quality of PROGRESS type 1 and 2 studies remains to be investigated. Monitoring of the quality of prognostic studies and their reporting will continue to be important to ensure improvement in the field. Ideally, a repository of critically appraised SRs of prognosis might provide a source of useful examples to guide future investigations.

### Additional file

Additional file 1: PRISMA checklist. (PPTX 53 kb)

## Appendix

### Literature search

The following search string was used: (“Circulation” [Journal] OR “BMJ (Clinical research ed.)” [Journal] OR “Annals of internal medicine” [Journal] OR “Stroke; a journal of cerebral circulation” [Journal] OR “JAMA: the Journal of the American Medical Association” [Journal]) AND ((“2000/01/01” [PDAT]: “2012/12/31” [PDAT]); the search was performed with and without ANDing the term “review” [Publication Type]).

## Abbreviations

CI: Confidence interval
MOOSE: Meta-analysis of Observational Studies in Epidemiology
NA: Not applicable
PRISMA: Preferred Reporting Items for Systematic reviews and Meta-Analyses
SD: Standard deviation
SR: Systematic review
